# Microtensile Bond Strength of Etch-and-Rinse Adhesives in Different Hydroabrasion Conditionings

**DOI:** 10.1155/2021/6649578

**Published:** 2021-02-17

**Authors:** Michele Tepedino, Maciej Iancu Potrubacz, Antonella Imperiale, Claudio Chimenti, Mario Capogreco, Maurizio D'Amario

**Affiliations:** ^1^Department of Biotechnological and Applied Clinical Sciences, University of L'Aquila, L'Aquila, Italy; ^2^Department of Life, Health and Environmental Sciences, University of L'Aquila, L'Aquila, Italy

## Abstract

The aim of the present study was to in vitro evaluate the effect of dentin conditioning with hydroabrasion on the microtensile bond strengths of three adhesive systems, compared to the standard etch-and-rinse technique. Sixty extracted human third molars were collected, and their midcoronal occlusal dentin was used for the microtensile bond strength test. Teeth were randomly assigned to three groups according to the adhesive system used: ExciTE F DSC, ENA Bond, and Scotchbond Universal. Specimens from each group were further divided into four subgroups: control specimens were treated with standard adhesive procedures; hydroabraded (HA) specimens were subjected to preventive hydroabrasion with three different intensity levels. After bonding procedures, composite crowns were incrementally built up. After thermocycling, specimens were subsequently sectioned into 1 × 1 mm sticks, and microtensile bond strengths were measured. Data were statistically analyzed. Failure mode analysis was performed. There were no significant differences in terms of bond strength between standard adhesion protocols and adhesion with HA preconditionings. On the other hand, the type of adhesive used had a significant effect on the tensile bond strength. Subgroups treated with hydroabrasion at higher intensity showed a slightly increased frequency of cohesive fractures. In conclusion, hydroabrasion can be used for dentin cavity preparation or finishing, since it does not seem to affect the bonding effectiveness.

## 1. Introduction

Adhesive restorative dentistry is an area of great interest for both research and clinical practice, and new materials and clinical strategies are continuously developed to restore the structural and aesthetic integrity of the damaged teeth [1, 2]. Basically, the bond with dental tissues is based on an exchange process in which minerals removed from the dental hard tissues after acid conditioning are substituted by resin monomers that become micromechanically interlocked in the porosities upon polymerization [3]. Current adhesion approaches differ on how dental adhesives interact with the smear layer and are grouped into two basic groups: etch-and-rinse (ER) and self-etch (SE) adhesives. ER adhesives involve complete elimination of the smear layer and superficial hydroxyapatite through etching with a separate acid gel (usually, phosphoric acid) [3] followed by infiltration of adhesive monomers that permeate the microporosities forming hybrid tissue known as the resin-dentin interdiffusion zone or “Hybrid Layer” [4, 5]. In contrast, SE adhesives make the smear layer permeable without totally removing it. This does not require a separate phosphoric acid-etch step as acidic adhesive monomers are utilized to partially dissolve the smear layer and demineralize the underlying dentin/enamel while infiltration is achieved simultaneously [6].

The recent introduction of universal adhesives, which contain acidic functional monomers, such as 10-methacryloyloxydecyl dihydrogen phosphate (MDP), has allowed clinicians to simplify the utilization of adhesives [7, 8]. A systematic review on universal adhesives [9] reported that dentin bond strengths of these adhesives appear not to be influenced by the bonding strategies employed, suggesting that universal adhesives should be safely used in an ER mode. Nevertheless, achieving an effective bond between restorative materials and dentin still represents a major challenge. This is due to the characteristic nature of the dentin, which shows a conspicuous organic content and dentinal fluid in its tubules. Another factor that negatively affects adhesion is the presence of a smear layer on its surface resultant from the use of rotating tools [10–12]. To improve the interaction between resin and dentin and with the aim of removing debris that could impair the final bonding restoration, different dentinal pretreatment techniques have been proposed over time [13–15]. Considering only mechanical techniques, it is worth mentioning the use of air-abrasion particles (APAs) that allows the improvement of dentin bonding through an ultrafine mechanical retention [16–18]. APA is a dentinal pretreatment technique based on the use of aluminium oxide particles carried by compressed air [19–21], and several experimental studies have identified the effectiveness of this technique in improving the bond strength by roughening the dental surface, thus increasing the contact area with the adhesive system [16, 17]. In addition, Rafael et al. reported that APA, while producing dentin abrasion, also preserves the original diameter of the dentinal tubules' orifice and the amount of intertubular dentin [18].

The hydroabrasion (HA) concept has been introduced to make easier the application of traditional abrasion. In the HA process, the aluminium oxide particles are accelerated by air and water spray. This method offers several advantages, improving the comfort for both the operator and the patient by limiting the diffusion of powder particles around the operative field, keeping the site clean from additional debris, moisturizing the tooth, and keeping the dentinal fluid inside the tubules, reducing sensitivity [22, 23]. In addition, HA can be used for hard tissue removal, substituting rotary instruments, achieving cavity preparation, and dentin conditioning in a single operative phase [23]. Despite all these potential advantages, there is no information regarding the effects of this procedure on the bond strength of different adhesives to human dentin. Indeed, to the best of our knowledge, HA was previously investigated only as a method for composite removal after orthodontic debonding [24], but there are no studies evaluating the bond strength after dentin conditioning with such technique.

The aim of this in vitro study was, therefore, to investigate the effect of HA conditionings, with different levels of air pressure and particle flow, on the microtensile bond strengths of three commercial adhesives used with ER technique. The null hypotheses were that no difference exists in terms of bond strength between (1) the standard ER adhesion protocol and adhesion with HA preconditionings and (2) the three tested adhesives.

## 2. Materials and Methods

### 2.1. Specimen Preparation

This study used deidentified extracted human molar teeth. To comply with research ethics, the study protocol was reviewed and approved by the Internal Review Board of University of L'Aquila (Protocol number: 25403). All patients provided informed consent, and all methods were performed in accordance with the relevant guidelines and regulations.

Sixty third molars extracted for therapeutic reasons were collected; to be included in the study, teeth were observed under 2.5X magnifying loupes and had to present an intact enamel surface and have no signs of erosion or abrasion, no surface demineralization, no decay, and no traumatic damage provoked by forceps during the extraction procedure. The teeth were rinsed with water and stored before the start of the investigation in a 0.5% chloramine-T solution at 4°C for not more than one month. The outer part of the crown of the selected dental specimens was removed perpendicularly to its longer axis using a low-speed diamond saw (Micromet M, Remet, Bologna, Italy) under copious water spray to expose as much dentin as possible while maintaining the integrity of the pulp chamber. The exposed dentin surface was ground with the 180-grit silicon carbide (SiC) abrasive paper on a polisher (Polimet, Buehler Ltd., Lake Bluff, IL, USA) for 30 s under abundant running water to create a thin layer of the standardized smear layer on the dentinal surface.

The bonding surfaces were then examined under a stereomicroscope (Nikon SMZ10, Tokyo, Japan) to ensure that they were free from residual enamel. If any enamel remained, the surface was ground again until all enamel was removed. Teeth were then randomly divided into three groups (*n* = 20 for each group), according to the adhesive system tested (Table 1).

Subsequently, dental specimens were randomly divided into four subgroups, which were subjected to different pretreatment protocols (5 teeth/subgroup):

(i) Control group (C), treated with adhesive protocol by the respective manufacturers (Table 1), without any other pretreatment
(ii) Hydroabraded groups (HA), subjected to preventive hydroabrasion and subsequent application of the two-step adhesive system. To evaluate the effect of three different cutting intensities (minimum, average, and maximum), three different HA groups were exposed to HA with different conditions of air pressure and flow intensity of aluminium oxide particles, as follows:
  - HA/3 bar group, hydroabrasion conditioning with an air pressure of 3 bar and minimal aluminium oxide particle flow
  - HA/5 bar group, hydroabrasion conditioning with an air pressure of 5 bar and medium aluminium oxide particle flow
  - HA/7 bar group, hydroabrasion conditioning with an air pressure of 7 bar and maximum aluminium oxide particle flow

HA procedures were performed using an intraoral sandblaster (PrepStart H2O, Danville Materials Inc., San Ramon, CA, USA), using a powder of 50 *μ*m aluminium oxide particles (Danville Materials Inc., San Ramon, CA, USA). The studied HA system is designed for a wide range of applications, from superficial stains removal to enamel cutting and cavity preparation, but a detailed protocol is not present in the current literature. Therefore, to evaluate a range of achievable effects, three levels of sandblasting intensity were arbitrarily chosen by regulating the air pressure and the aluminium particle flow. The sandblasting was performed for 10 s holding the nozzle at a distance of 5 cm from the dental surface, with an angulation of approximately 90°. Dentin surfaces were then rinsed with water for 15 s and dried for 5 s.

All specimens from the control and HA groups were then etched for 15 seconds with the phosphoric acid gel provided by the respective manufacturers (Table 1) and were subsequently rinsed using a water spray for at least 15 seconds. Excess water was blot-dried from the dentin surface with a wet cotton pellet, leaving the surface visibly moist.

In each group, the adhesive system was applied according to the manufacturers' instructions (Table 1) and then light-cured for 20 seconds with a curing light (Bluephase C8, with 800 mW/cm^2^ output, Ivoclar Vivadent AG, Schaan, Liechtenstein).

Following the adhesive procedures, a composite resin (Aura Ultra Universal Restorative Material; SDI, Victoria, Australia; batch no: 140758) was applied on all prepared dentinal surfaces, reaching a thickness of 4 mm through 1 mm increments. Each composite increase was light-cured for 40 seconds. After the restorative procedures described above, the specimens were stored in distilled water at 37°C for 24 h and then underwent 30,000 thermal cycles in deionized water from 5°C to 55°C, with a 30-second dwelling time and 5-second transfer between temperature baths (LTC100, LAM Technologies Electronic Equipment, Firenze, Italy) [25, 26].

Specimens were then embedded into resin blocks (Orthojet, Lang Dental Mfg, Inc., USA). The blocks were then sectioned perpendicularly to the adhesive interface with a diamond saw (Micromet M, Remet; Bologna, Italy) under cooling/lubrication to produce beams with an adhesive area of approximately 1 mm^2^. Six beams from the central part of each specimen were obtained per tooth. A total of 30 beams (*n* = 30) were subsequently used for each subgroup. The microtensile bond strength test was performed for each beam.

### 2.2. Microtensile Bond Strength Test

Beams were fixed on metal support plates, with cyanoacrylate cement, used in a universal testing machine (LTM 150; LAM Technologies Electronic Equipment) connected to a computer. Each stick was stressed until failure, with a cross-head speed of 0.5 mm/min. A two-link chain was interposed between the device and the upper clamp of the testing machine. Once tested, specimens were removed from the testing devices, and the cross-sectional areas of the fracture sites were measured with a digital caliper (series 500 Caliper; Mitutoyo America, Aurora, IL, USA) to calculate the ultimate tensile bond strength expressed in MPa.

The postfracture sticks were then observed by the scanning electron microscope (SEM) (EVO 50 XVP LaB6, Carl Zeiss, Cambridge, UK) at 100x or higher magnification to determine the fracture modes, which were divided as follows: type 1, adhesive fracture between adhesive agent and dentin; type 2, adhesive fracture between adhesive agent and dentin plus partial cohesive fracture in the composite restoration or dentin (mixed failure); type 3, cohesive fracture in dentin; and type 4, cohesive fracture in the composite restoration [16, 27].

### 2.3. Statistical Analysis

Descriptive statistics were calculated for microtensile strength within all groups. Data distribution and homoscedasticity were tested with the Shapiro–Wilk normality test and the Levene test, respectively. To evaluate the effects of adhesive type and conditioning treatment on microtensile strength values, a linear mixed model was used. Adhesive type and the conditioning method were considered as fixed effects, while the origin of different beams from the same tooth was considered as a random effect, to account for any possible cluster effect. If statistically significant effects were found, pairwise comparisons were performed using Tukey's honestly significant difference (HSD) test. First type error was set as *p*=0.05. Statistical analysis was performed using SPSS software (Statistical Package for Social Sciences for Windows v.26, IBM Corp, Armonk, NY, USA).

## 3. Results

Descriptive statistics of microtensile strength values for different combinations of adhesives and treatment used, as well as the results from the normality test, are reported in Table 2. No pretest failure was registered. The Levene test revealed that the error variances were not equally distributed (*F* = 4.53, *p* < 0.001), but since the linear mixed model is robust to heteroscedasticity [28], it was run despite this finding. Regarding the results of the linear mixed model, there was no significant interaction between the effects of adhesive type and conditioning treatment on microtensile strength (Table 3). The null hypothesis that no difference exists in terms of bond strength between the standard adhesion protocol and the adhesion with HA preconditioning was thus accepted. On the other hand, microtensile strength was significantly different between the three adhesives used, regardless of the conditioning treatment (Table 4). The second null hypothesis was thus rejected. The random effect had no significant result (estimate of variance <0.001).

**Figure 1:**
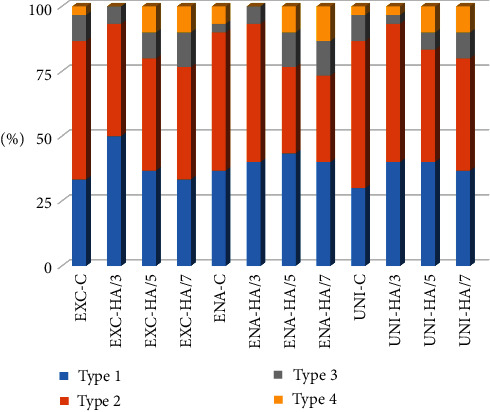
Distribution (%) of failure mode in experimental groups after the microtensile bond strength test. Type 1, adhesive fracture between adhesive agent and dentin; type 2, adhesive fracture between adhesive agent and dentin plus partial cohesive fracture in the composite restoration or dentin (mixed failure); type 3, cohesive fracture in dentin; and type 4, cohesive fracture in the composite restoration. EXC, ExciTE F DSC; ENA, ENA Bond; UNI, Scotchbond Universal; C, control group; HA/3, hydroabrasion group at 3 bar; HA/5, hydroabrasion group at 5 bar; HA/7, hydroabrasion group at 7 bar.

**Figure 2:**
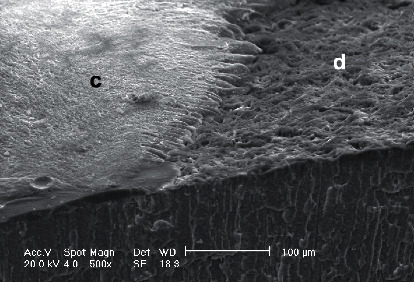
Scanning electron microscope (SEM) image of a type 2 failure (adhesive fracture between adhesive agent and dentin plus partial cohesive fracture in the composite restoration) (PB-A). c, composite; d, dentin. Magnification ×500.

Figure 1 displays the results of the failure mode analysis. Types 1 (adhesive fracture between adhesive agent and dentin) and 2 failures (adhesive fracture between dentin and adhesive agent plus partial cohesive fracture in dentin or composite restoration) were the most prevalent failure modes in all subgroups (Figure 2). The other types of failure modes were relatively uniform among all subgroups. HA group 2 and HA group 3 showed a slightly greater number of cohesive fractures (types 3 and 4).

## 4. Discussion

The aim of this experimental study was to investigate the effect of HA through the use of 50 *μ*m aluminium oxide particles emitted by air and water spray, on the tensile bond strength of three ER adhesive systems. The microtensile bond test is a valuable tool for such scope, and its technique is being codified and validated by a large number of studies [29]. The extraoral storage time of the collected teeth does not influence bonding strength, as demonstrated by previous studies [30].

Regarding sample preparation, the use of abrasive papers allowed the creation of a standardized smear layer, more uniform and with less irregularities compared to the use of rotating instruments [31]. Artificial aging is known to influence the results of microtensile bond tests [26]; therefore, the use of 30,000 cycles of thermocycling aging was chosen and considered adequate because previous reports showed that a shorter cycling had no appreciable effect on most specimens [16, 26, 32].

The use of HA as a dentin pretreatment technique in this study has its rationale in some experimental studies that involved air polishing for the same purpose [33–35]. Moreover, the HA system used in the present study has been designed to be used also for cavity preparation. Therefore, in a clinical environment, such HA treatment can be either for dentin conditioning or the result of hard tissue removal for cavity preparation. Indeed, air polishing has been investigated for its alleged ability to increase the bond strength between dentinal tissue and restorative material [35] by removing the smear layer and roughening the dental surface [36]. The results reported in the literature are still controversial, principally because the influence of air polishing on the adhesive strength bond depends on the type of powder and the adhesive system used. One of the most influential studies on this subject, performed by Nikaido et al. [34], reported that the action of air polishing might cause superficial maceration of the collagen fibres on the dentin surface. This could bring about a decrease in adhesive bond strength for those systems that contain only an acidic primer for dentin conditioning because of the formation of an unsuitable hybrid layer. Moreover, according to the experimental work of Frankenberger et al. [35], air polishing with sodium bicarbonate particles induces damage to the dentinal tissue and reduces the bonding force regardless of the adhesive strategy. On the other hand, the work of Tamura et al. [37] considered the possibility of evaluating the influence of air polishing on the bond strength by comparing two powders (glycine and sodium bicarbonate) and various universal adhesive systems used in the SE mode. The results reported that the sodium bicarbonate particles decrease the adhesive bond strength, unlike the glycine particles, which positively influences it. These data were justified by an SEM observation that identified the removal of the smear layer in the samples treated with glycine and the persistence of the smear layer in the samples treated with sodium bicarbonate.

Moreover, dentinal pretreatment through sandblasting is able to induce an improvement of adhesive bond strength by increasing micromechanical retention and tissue's wettability [38]. This hypothesis has been corroborated by some recent works. Mujdeci and Gokay [17] tested the abrasive effect of sandblasting associated with various restorative materials on enamel and dentin. This study recorded an increased strength bond for all samples tested. This result was associated with two conditions induced by the examined technique: greater contact area between the material and the dental tissue and the increased wettability of the structure. Rafael et al. [18], through their evaluation of the sandblasting's effect by SEM, showed that the increase in the contact surface available for adhesion is linked to the greater exposure of the tubular orifices and to the greater irregularity of the intertubular dentinal surface. Additionally, the experimental study carried out by D'Amario et al. [16] in 2017 with the aim of testing the effectiveness of dental sandblasting in relation to four different ER adhesive systems confirmed the previous findings; the tensile test detected an increasing variation in the values of the adhesion force in all sandblasted groups and the prevalence of cohesive type of bond failure.

The results of the present investigation showed a nonstatistically significant incremental variation of the strength bond values comparing the HA groups to the control group, and this is in agreement with other previous studies [39]. The first null hypothesis was thus accepted.

SEM evaluation of dentinal surfaces conditioned with the air-abrasion system revealed a roughened surface, with partially opened tubules and preserved peritubular and intertubular dentin [39–41], while other studies reported an even larger amount of debris with totally occluded tubules [42]. When using an ER adhesive system, the rinsing procedure removes the smear layer; therefore, its properties are not relevant in determining the final bonding strength [31]. On the other hand, an increased roughness of the dentinal surface could improve the bonding strength by increasing the contact surface and its wettability, but some studies concluded that air-abrasion preparation of dentinal surfaces did not result in an augmented adhesion [39, 43, 44]. In contrast, Sinjari et al. [45] reported an increased bonding strength with an ER system after air-abrasion pretreatment, but the authors did not specify the type of sandblasting performed and used bovine teeth, which have slightly different properties from human teeth [28]. Based on those observations, some authors concluded that surface roughness is not the main factor in dentinal adhesion, which is also influenced by the physical and chemical characteristics of the dentin surface [39]. These results cannot be directly compared to those of the present study because the hydroabrasion technique used here is a different method than the previously studied air abrasion, and this is the first report of this kind in the literature. Therefore, future studies should aim to disclose any difference between the two methods. In addition, the hydroabrasion device used in the present study is designed for cavity preparation and can be used as an alternative to rotating instruments rather than just as a surface conditioning. Moreover, the novelty to be proposed in this experimental study is associated with further advantages that wet sandblasting could bring, including in particular, a better comfort both for the operator and for the patient linked to the presence of water, which avoids the spread of the particulate, cleaning of the dentinal surface from additional debris associated with the pretreatment itself, the hydration of the dentinal tissue, and the protection of the fluids inside the open dentinal tubules, minimizing the dentinal sensitivity.

All the evaluated adhesive systems showed acceptable bond strength values. Nevertheless, UNI exhibited the highest bond strength results, regardless of the dentin pretreatment protocol. The second null hypothesis was that the adhesive system would make no difference to the bond strength to dentin and was thus rejected. UNI contains the phosphate acid monomer, 10-MDP (10-methacryloyloxydecyl dihydrogen phosphate), which chemically bonds to hydroxyapatite, forming hydrolytically stable calcium salts in the form of “nano-layering” on hydroxyapatite [46]. Additionally, UNI contains a polyalkenoic acid copolymer in its composition, which interacts with apatite substrates following the same adhesion-decalcification reaction [47]. Thus, for UNI, both bonding mechanisms promoted micromechanical retention by diffusion of resin monomers and chemical adhesion. In fact, both clinical [48] and in vitro [46, 49] studies found that there was no statistical difference among bonding strategies when a universal adhesive was used on dentin and that etching had only a minor effect with universal adhesives. However, the no application of acid etching of dentin allows chemical bonding between the functional monomers and the dentin hydroxyapatite. For this reason, the supposed benefits of HA procedures could be more pronounced for self-etching adhesives, as acid etching always completely removes the smear layer. In future studies, it would be very interesting to evaluate the effect of each HA treatment on dentin surface, before and after acid etching, and to test the effect of hydroabrasion using SE adhesives.

Regarding the limitations of the present study, the possible adverse effects of hydroabrasion on the dentin surface were not investigated. In addition, the possible influence of pulpal pressure on bonding strength was not taken into consideration in the present experimental setup, although some researchers [50] reported that artificially simulated pulpal pressure does not influence the results of microtensile bond strength.

## 5. Conclusions

It is possible to conclude that hydroabrasion with 50 *μ*m aluminium oxide does not seem to significantly increase or impair the microtensile strength values of different adhesives used with ER strategy in dentin; therefore, it could be used for cavity preparation instead of rotating instruments.

## Figures and Tables

**Table 1:** Manufacturer, composition, and application mode of the adhesive systems tested.

Adhesive	Batch no.	Composition	Phosphoric acid gel	Application mode	Manufacturer
ExciTE F DSC (EXC)	V49576	HEMA, dimethacrylate, phosphonic acid acrylate, highly dispersed silicone dioxide, initiators, stabilizers, and potassium fluoride in an alcohol solution.	Total Etch (37 wt.% phosphoric acid gel)	ExciTE F DSC was applied with light brushing motion for 15 seconds and then air dried for 5 seconds and dispersed to a thin layer with a weak stream of air.	Ivoclar Vivadent AG, Schaan/Liechte nstein
ENA Bond (ENA)	2019010066	Modified acrylate acid, polyacrylate acid, methacrylate, ethyl alcohol, catalysts, and stabilizers.	Ena Etch (37 wt.% phosphoric acid gel)	EnaBond was applied on the dentin surface, following the wet technique, with a microbrush, gently rubbed for 20–30 s, then distributed with an air spray for at least 15 s at a distance of 10 cm to form a slightly shiny adhesive film.	Micerium, Avegno, Genova, Italy
Scotchbond Universal (UNI)	6450964	10-MDP, HEMA, silane, dimethacrylate resins, Vitrebond copolymer, filler, ethanol, water, and initiators	Scotchbond Universal Etchant (32 wt. % phosphoric acid gel)	Scotchbond Universal was applied, following the wet technique, to the prepared dentin surface and scrubbed in for 20 seconds, and then gently air dried for approximately 5 seconds to evaporate the solvent.	3M Oral Care, Seefeld, Germany

**Table 2:** Descriptive statistics for microtensile strength of different groups with different dentin treatment.

Adhesive	Treatment							
	Control		HA/3		HA/5		HA/7	
	Mean ± SD^*∗*^	Normality test^*∗∗*^	Mean ± SD	Normality test^*∗∗*^	Mean ± SD	Normality test^*∗∗*^	Mean ± SD	Normality test^*∗∗*^
EXC (*n* = 120)	27.7 ± 10.8	0.100	27.9 ± 6.3	0.015	28.5 ± 7.9	0.003	30.6 ± 11.9	0.014
ENA (*n* = 120)	32.5 ± 6.4	0.015	30.7 ± 6.4	0.028	31.1 ± 6.6	0.451	32.9 ± 7.4	0.001
UNI (*n* = 120)	33.9 ± 4.9	0.141	34.2 ± 4.9	0.080	35.1 ± 5.9	0.012	34.8 ± 6.2	0.021

^*∗*^Mean ± standard deviation in MPa. ^*∗∗*^*p* value from the Shapiro–Wilk normality test. HA/3, hydroabrasion conditioning with an air pressure of 3 bar and a minimum aluminium oxide particle flow; HA/5, hydroabrasion conditioning with an air pressure of 5 bar and a medium aluminium oxide particle flow; HA/7, hydroabrasion conditioning with an air pressure of 7 bar and a maximum aluminium oxide particle flow; EXC, ExciTE DSC, Ivoclar Vivadent AG, Schaan/Liechtenstein; ENA, ENA Bond, Micerium, Genova, Italy; UNI, Scotchbond Universal, 3M Oral Care, Seefeld, Germany.

**Table 3:** Linear mixed-effects model to determine the effect of adhesive and treatment on microtensile strength.

	Numerator d*f*	Denominator d*f*	*F*	*p*
Intercept	1	348	6523.7^*∗*^	<0.001
Adhesive†	2	348	18.4^*∗*^	<0.001
Treatment ‡	3	348	0.9	0.412
Adhesive^*∗*^Treatment	6	348	0.4	0.902

^*∗*^Statistically significant with *p* < 0.01; ^†^use of either a ExciTE, ENA Bond, or Scotchbond universal adhesive; ^‡^dentin treatment with only acid etching (control group), hydroabrasion at 3 bar with minimum aluminium oxide particle flow (HA/3), hydroabrasion at 5 bar with medium aluminium oxide particle flow (HA/5), or hydroabrasion at 7 bar with maximum aluminium oxide particle flow (HA/7).

**Table 4:** Tukey's honestly significant difference post hoc test for the effect of adhesive on microtensile strength.

Adhesive	Mean difference	Standard error	*p*	95% confidence interval	
				Lower bound	Upper bound
EXC vs. ENA	−3.1^*∗∗*^	0.96	0.004	−5.4	−0.9
EXC vs. UNI	−5.8^*∗∗*^	0.96	<0.001	−8.1	−3.6
ENA vs. UNI	−2.7^*∗*^	0.96	0.014	−4.9	−0.4

^*∗*^Statistically significant with *p* < 0.05; ^*∗∗*^statistically significant with *p* < 0.01. EXC, ExciTE DSC, Ivoclar Vivadent AG, Schaan/Liechtenstein; ENA, ENA Bond, Micerium, Genova, Italy; UNI, Scotchbond Universal, 3M Oral Care, Seefeld, Germany.

## Data Availability

No data were used to support this study.
